# Characterization of DNA Methylation Episignatures for Radon-Induced Lung Cancer

**DOI:** 10.3390/ijms26146873

**Published:** 2025-07-17

**Authors:** Ziyan Yan, Huixi Chen, Yuhao Liu, Lin Zhou, Jiaojiao Zhu, Yifan Hou, Xinyu Zhang, Zhongmin Chen, Yilong Wang, Ping-Kun Zhou, Yongqing Gu

**Affiliations:** 1 Beijing Institute of Radiation Medicine, Beijing 100850, China; yanziyan777@163.com (Z.Y.); yuhaoliu97@163.com (Y.L.); zhoulin141113@163.com (L.Z.); zhu141113@163.com (J.Z.); nkwangyilong@126.com (Y.W.); 2 Hengyang Medical College, University of South China, Hengyang 421001, China; 18173354730@163.com; 3 College of Life Sciences, Hebei University, Baoding 071001, China; hyfaxx15633798061@163.com; 4 School of Public Health, University of South China, Hengyang 421001, China; zxy202507@163.com; 5 PLA Rocket Force Characteristic Medical Center, Beijing 100850, China; hero_czhm@163.com

**Keywords:** radon, lung cancer, DNA methylation, episignatures, RRBS, MassArray

## Abstract

Radon (Rn) exposure has a strong association with lung cancer risk and is influenced by epigenetic modifications. To investigate the characterization of DNA methylation (DNAm) episignatures for radon-induced lung cancer, we detected the specific changes in DNAm in blood and lung tissues using reduced representation bisulfite sequencing (RRBS). We identified the differentially methylated regions (DMRs) induced by radon exposure. The bioinformatics analysis of the DMR-mapped genes revealed that pathways in cancer were affected by radon exposure. Among them, the DNAm episignatures of MAPK10, PLCG1, PLCβ3 and PIK3R2 were repeated between lung tissue and blood, and validated by the MassArray. In addition, radon exposure promoted lung cancer development in the genetic engineering mouse model (GEMM), accompanied by decreased MAPK10 and increased PLCG1, PLCβ3, and PIK3R2 with mRNA and protein levels. Conclusively, radon exposure significantly changes the genomic DNAm patterns in lung tissue and blood. The DNAm episignatures of MAPK10, PLCG1, PLCβ3 and PIK3R2 have a significant influence on radon-induced lung cancer. This brings a new perspective to understanding the pathways involved in radon-induced lung cancer and offers potential targets for developing blood-based biomarkers and epigenetic therapeutics.

## 1. Introduction

Radon and its daughters are the largest source of natural radiation to humans. During its decay, radon releases alpha particles and generates high-LET radiation. It is identified as a Group I carcinogen by the International Agency for Research on Cancer (IARC), indicating potential carcinogenic effects on humans [1]. Predominantly found in various types of rocks and soils, radon gas can accumulate considerably in enclosed locations such as underground mines, basements and caves [2]. Data reveal that the radon levels in 15% of non-uranium mines in China exceed the national benchmark of 1000 Bq/m^3^, with some mines reaching over 10,000 Bq/m^3^ [3]. Epidemiological studies on occupationally exposed populations such as uranium miners have revealed links between radon exposure and increased lung cancer risk [4,5]. Subsequently, multiple case–control research efforts in Europe, China, and North America have detected carcinogenic impacts in the broader population [6,7,8]. These researchers noted a direct and significant 16% elevation in lung cancer risk for each 100 Bq/m^3^ increase in radon concentration [8]. As such, radon exposure has become a critical global public health issue.

Lung cancer ranks among the most common and lethal cancers globally, claiming approximately 1.8 million lives annually. Non-small cell lung cancer (NSCLC) represents about 85% of these cases, while small cell lung cancer (SCLC) accounts for about 15% [9]. The World Health Organization attributes lung cancer primarily to smoking, and radon is considered the second most important factor after smoking [10]. There are many subtypes of lung cancer, including adenocarcinoma, squamous cell carcinoma, and large cell carcinoma [11]. Because of cost-effectiveness and potential harm, imaging techniques have not been adopted for population screening [12]. Considering the difficulty of obtaining tissue, blood is undoubtedly the most valuable alternative biological sample. Recent advancements in precision medicine and targeted treatment have led to significant improvements in lung cancer management. Nonetheless, the outlook for lung cancer patients remains bleak, especially at advanced stages, with a five-year survival rate of merely 20% [13]. Hence, the early detection of lung cancer can allow for effective intervention through surgery or local treatment to improve the prognosis.

DNA methylation (DNAm), an essential epigenetic regulatory mechanism, contributes to tumorigenesis through the aberrant hypermethylation of CpG islands, leading to silencing of tumor suppressor genes (TSGs). In lung cancer, genes such as RASSF1A, p16, and APC are more frequently hypermethylated in tumor tissues compared to normal tissues, with links to smoking history, histological type and prognosis [14,15,16]. For instance, RASSF1A hypermethylation occurs in over 70% of NSCLC cases, indicating its potential usefulness as a diagnostic biomarker [17]. Emerging techniques for early screening focus on identifying methylation markers in bodily fluids. Hypermethylation of SHOX2 and PTGER4 in sputum, blood, or bronchoalveolar lavage fluid achieves over 80% sensitivity for early NSCLC detection, particularly in CT-negative cases [18]. Analyzing the methylation of circulating tumor DNA (ctDNA), such as dynamic monitoring of SOX17 and TAC1, correlates with tumor burden and provides a non-invasive tracking option [19,20]. These findings indicate that certain methylation alterations can serve as critical biomarkers for early lung cancer diagnosis and screening, as well as potential targets for therapeutic strategies. There is an epidemiological link between radon exposure and lung cancer risk [21], although experimental animal evidence remains insufficient to support this connection.

Radon is recognized as a standalone risk factor for lung cancer, and epigenetic modifications are involved in environmental carcinogenesis, such as DNA methylation. However, there is a lack of studies on the association of the methylation of specific tissues (such as blood and lung), and the key epigenetic targets of radon-induced lung cancer are not clear. This study concentrates on (1) investigating, for the first-time, DNAm modifications linked to radon exposure in lung tissue; (2) assessing the relationship between lung-derived and blood-derived differentially methylated regions (DMRs); (3) identifying changes in the DNAm of genes related to cancer signaling pathways due to radon exposure; and (4) validating the regulatory effects in gene expression within the GEMM caused by radon exposure. Our findings suggest that blood methylation can accurately reflect epigenetic changes in lung tissue, supporting the development of non-invasive biomarkers. Four genes have been pinpointed as critical nodes in radon-induced carcinogenesis. Epigenetic mechanisms appear directly connected to altered signaling pathways. This insight enhances our comprehension of the molecular basis of radon-related cancer development, providing a novel trajectory for targeted epigenetic therapies and aiding the early detection of lung cancer through the identification of blood epigenetic markers.

## 2. Results

### 2.1. Blood and Lung-Derived DMRs of Mice Exposed to Radon

**Figure 1:**
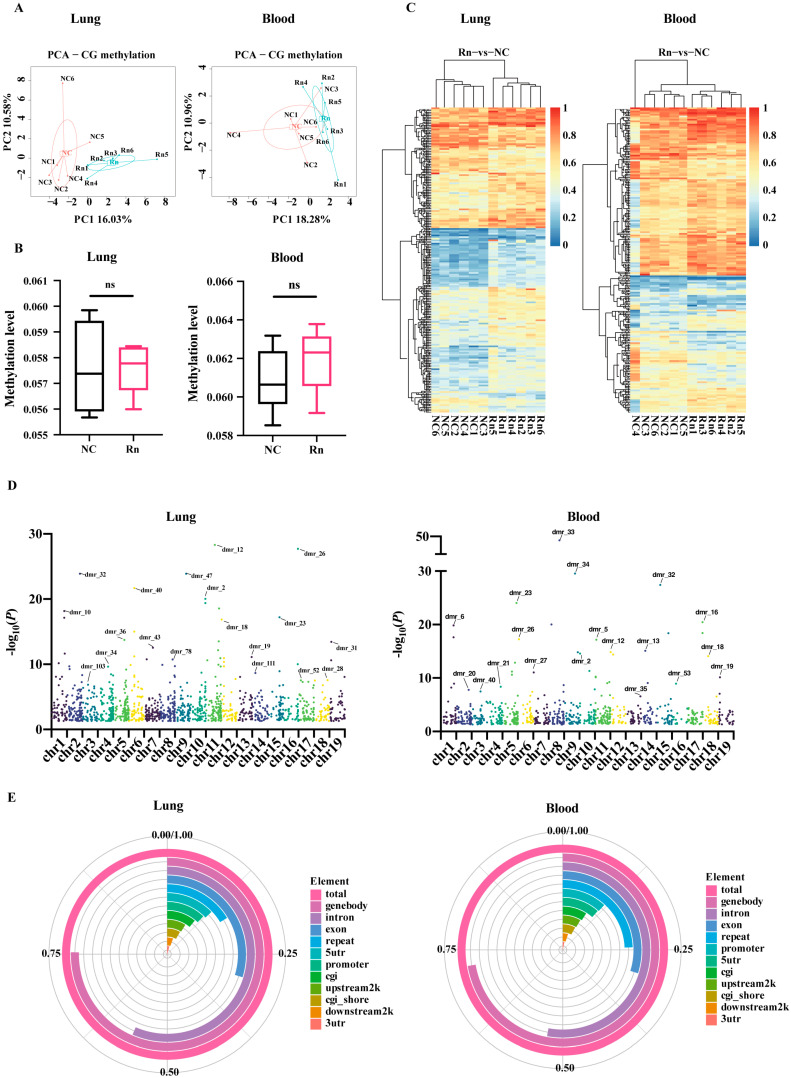
Blood and lung-derived DMRs of mice exposed to radon. (**A**) Principal component analysis (PCA) of DNA methylation loci. (**B**) Comparison of average methylation levels of standardized CpG sites between the exposure and the control group. *n* = 6 mice per group; ns, not significant. (**C**) Different DNA methylation loci presented are by the heatmap. (**D**) Manhattan plot of all DMRs on autosomal chromosomes. (**E**) Circos plots of the proportion of DNA methylation in the DMRs according to their distributions on genomic domains (promoter, genebody, repeat, upstream2k, 5′UTR, exon, intron, 3′UTR and downstream2k) and CpG context (CGI or CGI shore). NC, no-treatment control; Rn, radon exposure.

After data preprocessing, the exclusion of sex effects (X and Y chromosomes) and the removal of invalid values, CpG sites were obtained in 12 lung tissue and 12 peripheral blood samples for bioinformatics analysis. We used principal component analysis (PCA) to perform cluster analysis of lung and blood of mice in the normal group and the radon-exposed group, respectively, as shown in Figure 1A. It can be seen that DNAm levels in the lung tissue samples distinguished the two groups well, whereas the peripheral blood samples appeared to be less effective than the lung tissue samples. Lung tissue has better cell homogeneity, which can better capture the differences between groups, while peripheral blood is a mixed cell sample with large fluctuations in cell composition, and this heterogeneity dilutes the methylation signal of specific cell populations. The mean methylation levels of all CpG sites analyzed were not statistically different between the two groups (Figure 1B). Figure 1C demonstrates a heat map partitioned into two segments, wherein the radon-exposed group shows hypermethylation at CpG sites, contrasting with the hypomethylation observed in the control group. The Manhattan plot illustrates the widespread distribution of methylation sites across chromosomes 1 to 19, highlighting the most distinct methylation site markers on each chromosome in Figure 1D. Comprehensive details are listed in Tables S1 and S2. Figure 1E shows that blood- and lung-derived DMRs were distributed in similar proportions across genomic domains and CpG contexts, mainly in gene functional domains such as intron and exon promoters and the CpG island. These DMRs with different localizations may be involved in the regulation of gene expression induced by radon exposure through different mechanisms.

### 2.2. Regional Analysis of Hypomethylated and Hypermethylated DMRs

**Figure 2:**
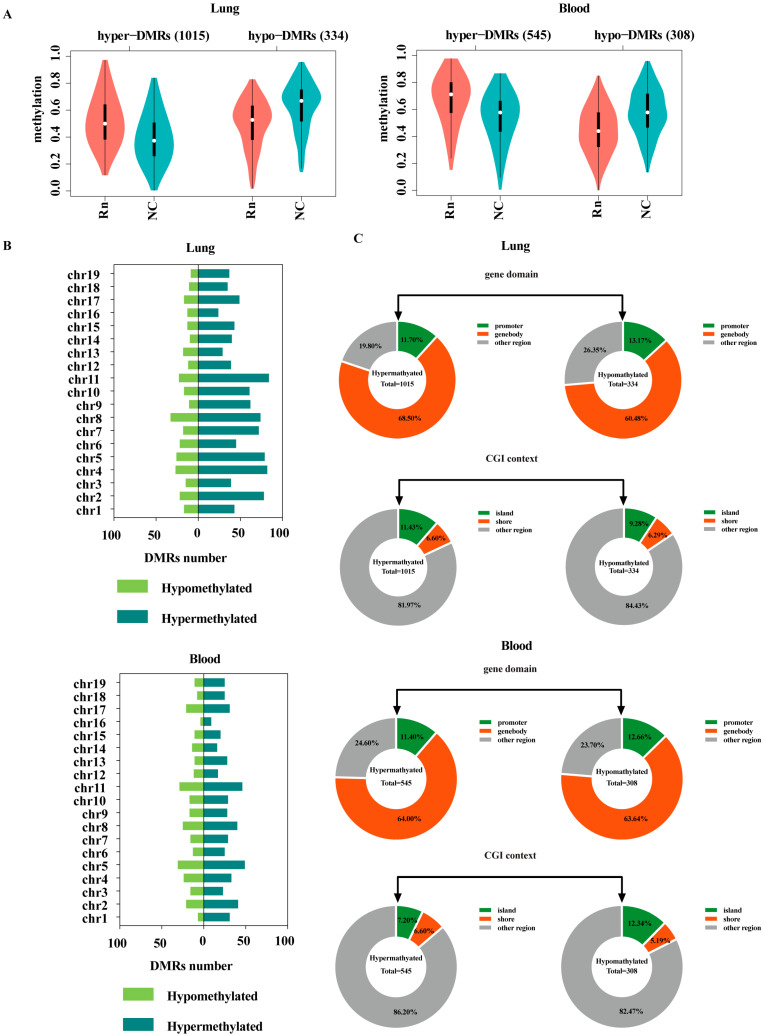
Regional analysis of hypomethylated and hypermethylated DMRs. (**A**) Violin plot of the distribution of DMR mean methylation levels. Hyper-DMRs refers to DMR that is hypermethylated in the Rn group, and hypo-DMRs represents DMR that is hypomethylated in the Rn group. (**B**) Distributions of hypomethylated and hypermethylated DMRs across chromosome 1 to 19. (**C**) Donut charts of DNA methylation percentages of hypomethylated and hypermethylated DMRs based on genomic domains and CpG context distributions. NC, no-treatment control; Rn, radon exposure.

The direction of DNAm (DNA hypermethylation or hypomethylation) is related to gene expression. There were a total of 1349 DMRs in the lung tissues from radon exposed mice, of which 1015 (75%) were hypermethylated and 334 (25%) were hypomethylated (Figure 2A, left). A total of 853 DMRs were identified in the blood of radon-exposed mice, of which 545 (64%) were hypermethylated and 308 (36%) were hypomethylated (Figure 2A, right). Figure 2B shows the distribution of the number of hypermethylated and hypomethylated DMRs across the 19 chromosomes. Among lung-derived DMRs, hypermethylated DMRs were mainly enriched on chromosome 11 (*n* = 84), and hypomethylated DMRs were mainly enriched on chromosome 8 (*n* = 33). Peripheral blood-derived DMRs were mainly enriched on chromosome 5 (*n* = 80), among which 49 (61%) were hypermethylated DMRs and 31 (39%) were hypomethylated DMRs. Figure 2C shows the distribution analysis of hypermethylated and hypomethylated DMRs in genomic domains and CpG contexts. Distribution analysis of genomic domains showed that more than 60% of hypermethylated and hypomethylated DMRs from the lung tissues were located in the body coding region, and about 10% were located in the promoter region. According to the CpG environment distribution analysis, about 10% of the methylated DMRs and hypomethylated DMRs from the lung tissues were located in the CpG islands, about 6% were located in CpG shores and more were located in other regions such as shelves and the open sea. In addition, the distribution trend in the gene functional structure and CpG region between peripheral blood-derived DMRs and lung-derived DMRs was consistent.

### 2.3. Functional Enrichment Analysis Associated with DMR-Mapped Genes

**Figure 3:**
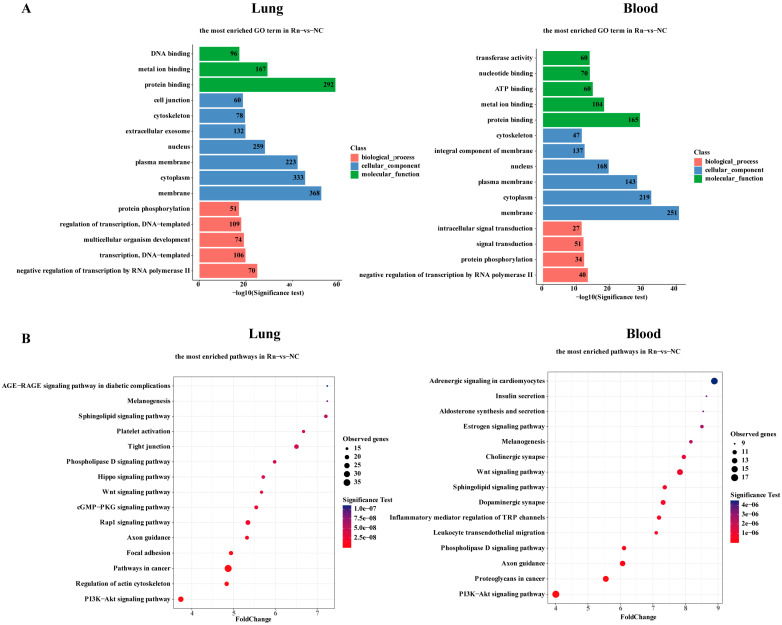
Functional enrichment analysis associated with DMR-mapped genes. (**A**) Top 15 significantly enriched GO terms of differentially methylated genes across biological process, cellular component, and molecular function. The ordinate representing GO terms and abscissa showing *p* value (−log_10_ scale). (**B**) Bubble chart for KEGG pathway analysis of differentially methylated genes, where the ordinate indicates pathway terms and abscissa shows gene ratio, circle size and color denote the number of genes involved and significance of pathway, respectively. NC, no-treatment control; Rn, radon exposure.

In assessing the annotation of DMRs in radon-exposed tissues, genes overlapping with DMRs in the genebody, as well as adjacent upstream and downstream regions, underwent gene ontology (GO) enrichment analysis, categorized into biological process (BP), cellular component (CC) and molecular function (MF). The top 15 most significant GO entries are shown in Figure 3A, and detailed GO term information is listed in Table S3. As the target organ of radon exposure, 1320 DMR-mapped genes in lung tissues were associated with various GO genes. The most significant BP genes were associated with transcription regulation (e.g., negative regulation of transcription by RNA polymerase II; transcription, DNA-templated; regulation of transcription, DNA-templated) and genes involved in growth and development, such as multicellular organism development. The most prominent in CC are nuclear localization genes related to gene expression and chromatin state and membrane localization genes related to intercellular communication. The most prominent in MF are genes involved in protein binding, metal ion binding, and DNA binding. Compared with lung tissue, there were fewer genes significantly associated with GO terms in blood (836), but most of the enriched items overlapped. In particular, BP was also significantly enriched in genes related to protein phosphorylation and signal transduction, and MF was significantly enriched in genes involved in metabolism and enzyme activity.

The KEGG database was used for signaling pathway analysis, and bubble plots graphically displayed the top 15 significant results in lung tissue and blood samples (Figure 3B). Detailed pathway information is listed in Table S4. Both lung-derived DMRs and blood-derived DMRs mapped genes enriched in tumor (e.g., PI3K-Akt signaling pathway and Hippo signaling pathway) and metabolism (e.g., Sphingolipid signaling pathway and phospholipase D signaling pathway). Notably, the largest number and most significant pathway enriched for Lung-derived DMR-mapped genes was pathways in cancer, which was also significantly enriched in blood, although not in the top 15 results. In addition to that, lung-derived DMR-mapped genes were also enriched in tumor cell structure and metastasis (such as tight junction, focal adhesion, axon guidance and regulation of actin cytoskeleton). Blood-derived DMR-mapped genes were enriched in immunity and inflammation (such as leukocyte transendothelial migration and inflammatory mediator regulation of TRP channels), endocrine (such as estrogen signaling pathway, adrenergic signaling in cardiomyocytes, insulin secretion and aldosterone synthesis and secretion), nerve signal transduction (such as dopaminergic synapse, cholinergic synapse and axon guidance) and other pathways.

### 2.4. DNA Methylation Changes and Association Analysis in Cancer-Associated Genes

**Figure 4:**
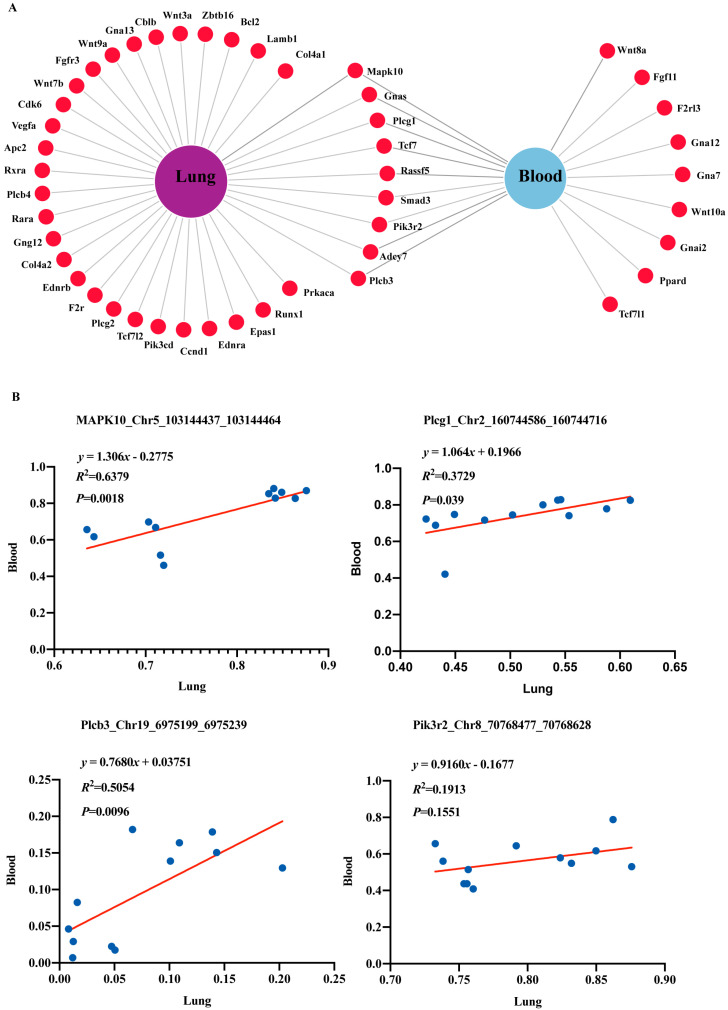
DNA methylation changes in cancer-related genes. (**A**) Venn network diagram showing overlapping cancer-related genes in lung and blood samples. (**B**) Scatter plots showing the correlation of methylation of target cancer-related genes in lung and blood samples.

During pathway analysis, pathways of cancer emerged in both the blood and lung tissue samples, which prompted us to further investigate the effects of radon exposure on these cancer-associated genes. Venn network diagram (Figure 4A) showed that there were twenty-eight lung tissue-specific cancer-associated genes, nine blood-specific cancer-associated genes, and nine overlapping lung tissue and blood cancer-associated genes. The common genes were TCF7, SMAD3, RASSF5, ADCY7, PIK3R2, MAPK10, PLCG1, GNAS and PLCβ3, as detailed in Table 1 and Table 2. We hypothesized that the differential methylation of these overlapping genes might serve as important DNAm episignatures for radon-induced lung cancer. By comparing the location and direction of DMR on genes, four DMRs with the same direction were obtained in both lung tissue and blood samples. Chr5_103144437_103144464 (annotated to MAPK10), Chr2_160744586_160744716 (annotated to PLCG1), and Chr19_6975199_697523 (annotated to PLCβ3) were hypermethylated. Chr8_70768477_70768628 (annotated to PIK3R2) was hypomethylated. There was a poor correlation between the mean methylation levels of lung and blood samples (*R*^2^ = 0.001239; *p* = 0.9135) (Figure S1A). We further analyzed the correlation between the DNAm levels of specific DMRs in lung tissue and blood samples. In Figure 4B, MAPK10_Chr5_103144437_103144464 (*R*^2^ = 0.6379; *p* = 0.0018), PLCG1_Chr2_160744586_160744716 (*R*^2^ = 0.3729; *p* = 0.039) and PLCβ3_Chr19_6975199_6975239 (*R*^2^ = 0.5054; *p* = 0.0096) showed a statistically significant linear correlation (*p* < 0.05) in lung tissue and blood samples, except for PIK3R2_Chr8_70768477_70768628 (*R*^2^ = 0.1913; *p* = 0.1551).

### 2.5. Validation of DNA Methylation in Cancer-Related Genes by MassArray

**Figure 5:**
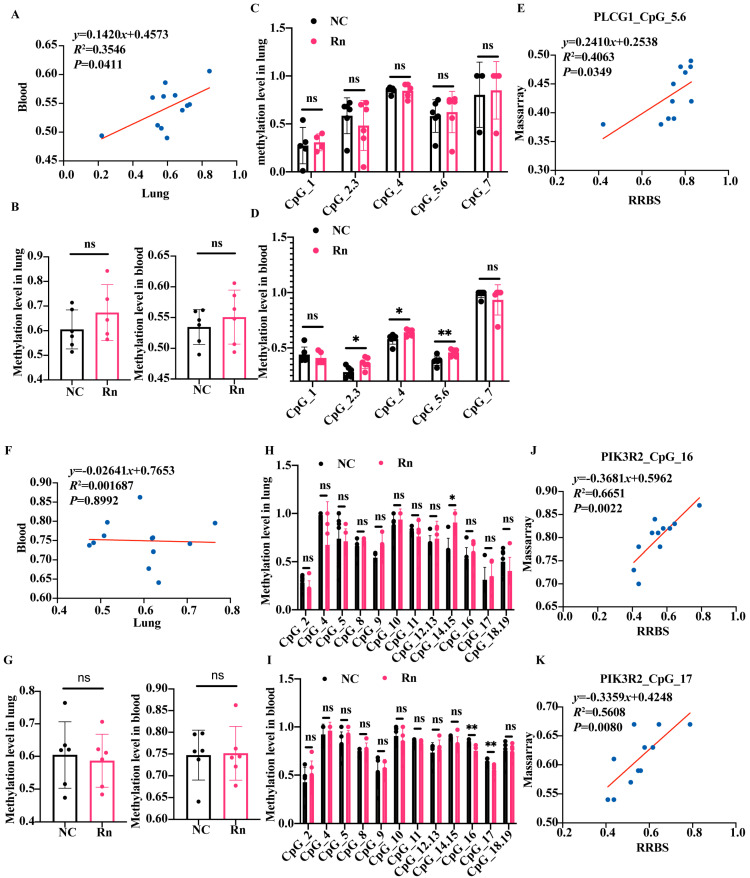
Validation of DNA methylation in cancer-related genes by MassArray. (**A**) Scatter plots showing the correlation of methylation of PLCG1 target region in lungs and blood. (**B**) The comparison of average methylation levels of all standardized CpG sites in PLCG1 target region between the exposure group and the control group. The comparison of methylation levels of each standardized CpG site in PLCG1 target region in lungs (**C**) and blood (**D**) between the exposure group and the control group. (**E**) Scatter plot shows the correlation of methylation level in PLCG1 target region in blood measured by RRBS (X axis, methylation level in PLCG1_Chr2_160744586_160744716) and MassArray (Y axis, methylation level in PLCG1 CpG 5.6 site). (**F**) Scatter plots showing the correlations of methylation of PIK3R2 target regions in lungs and blood. (**G**) Comparison of average methylation levels of standardized CpG sites within the PIK3R2 target region between the exposure and control groups, comparing individual CpG sites in lungs (**H**) and blood (**I**). (**J**,**K**) Scatter plots showing the correlation of blood methylation levels in PIK3R2 target regions measured by RRBS (X axis, methylation level in PIK3R2_Chr8_70768476_70768628) and MassArray (Y axis, methylation level in PIK3R2 CpG 16 or CpG 17 site). Individual dots represent the average methylation value at the specified site. *n* = 5–6 mice per group; * *p* < 0.05. ** *p* < 0.01. ns, not significant. NC, no-treatment control; Rn, radon exposure.

To further corroborate RRBS findings, the methylation levels covering all CpG sites within the gene’s target region were determined utilizing MassArray methylation analysis. The results showed that the mean methylation levels of the PLCG1 target regions were significantly consistent between lungs and blood (Figure 5A). The average methylation levels for PLCG1 target regions were significantly aligned between lungs and blood after radon exposure (Figure 5B). The PLCG1 target region contained seven CpG sites, of which methylation at CpG_2.3, CpG_4, and CpG_5.6 was significantly increased in blood but showed no difference in the lung samples (Figure 5C,D). Concordance was shown between the two assays for the methylation of three CpG sites on PLCG1 in blood, particularly CpG_5.6 (Figure 5E and Figure S1B,C). The mean methylation levels of the MAPK10 target regions showed poor agreement between lung and blood (Figure 5F). There was no difference in methylation levels in this region after radon exposure compared with controls (Figure 5G). The MAPK10 target region contained 19 CpG sites, among which the methylation of CpG_16 and CpG_17 sites was significantly reduced in the blood samples (Figure 5H,I), which strongly correlated with RRBS assay outcomes (Figure 5J,K).

### 2.6. Carcinogenic Effects of Radon and Cancer-Related Gene Expression in GEMM

**Figure 6:**
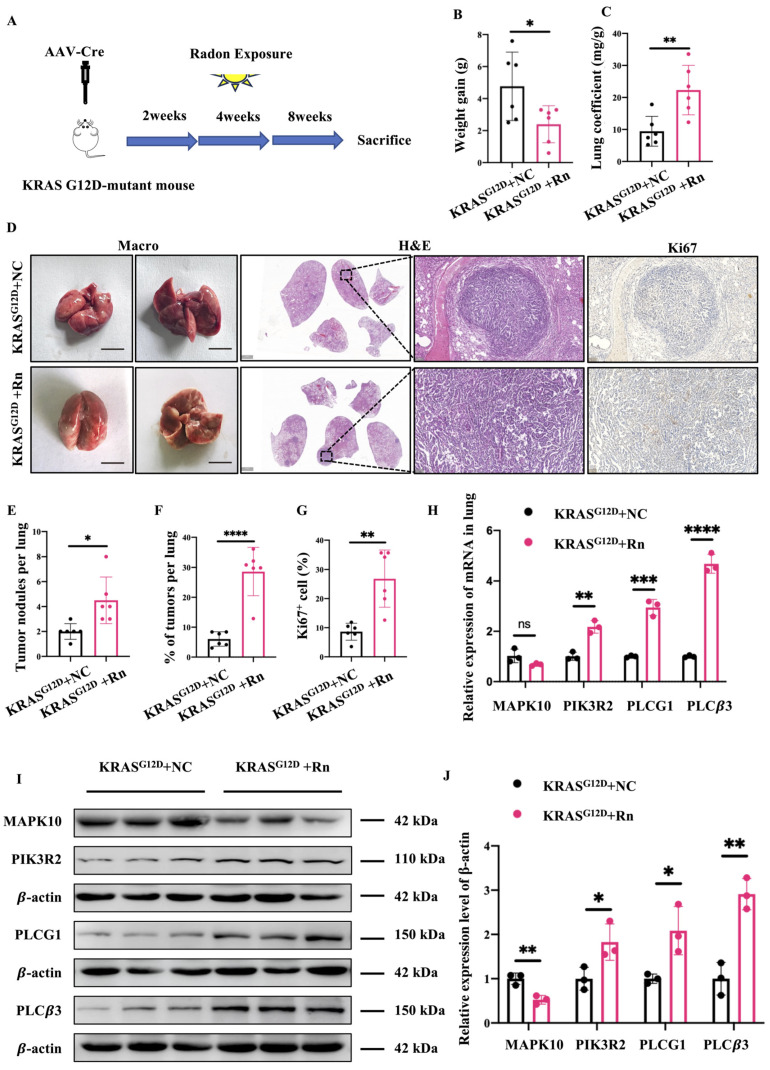
Carcinogenic effects of radon- and cancer-related gene expression in GEMM. (**A**) Schematic representation of the radon exposure protocol using the GEMM. Changes in the body weight (**B**) and lung coefficient (**C**) of mice 8 weeks after radon exposure (*n* = 6 mice per group). (**D**) Representative macroscopic lung images with H&E and IHC staining with the indicated antibodies of lung tissue sections from KRAS^G12D^ + NC and KRAS^G12D^ + Rn mice. Scale bars, 1 cm, 2000 μm or 100 μm. (**E**–**G**) Quantification of total number of tumor nodules (**E**), tumor burden (tumor area per lung; (**F**)), and proliferation (Ki67-positive cells per tumor area (**G**). *n* = 6 mice per group. (**H**) Gene expression normalized to β-actin measured by RT-qPCR. Three independent samples are shown for each group. (**I**) Western blots with the indicated antibodies of tumor lung biopsy lysate as indicated from mice. Three independent samples are shown for each group. (**J**) The expression of proteins in (**I**) was quantified. * *p* < 0.05. ** *p* < 0.01. *** *p* < 0.001. **** *p* < 0.0001. ns, not significant. NC, no-treatment control; Rn, radon exposure.

To explore the molecular dynamics mechanisms tied to radon-induced DNAm in lung tumorigenesis, we employed a genetically engineered mouse model (GEMM) for NSCLC, specifically mice with the KRAS G12D mutation (KRAS^G12D^ mice). The pattern of mice is shown in Figure 6A. First, the tracheas of KRAS^G12D^ mice were infected with the lentivirus-expressing CRE recombinase, and after 2 weeks, they were exposed to radon or a background environment for 4 weeks to observe tumorigenesis. At 16 weeks post-infection (12 weeks after the end of radon exposure), we observed a significant suppression of body weight and a significant increase in lung coefficients in radon-exposed KRAS^G12D^ mice compared with KRAS^G12D^ mice kept under control conditions (Figure 6B,C). Pathological staining (Figure 6D) showed that inflammatory cell infiltration was evident in the lung structure, and there were obvious masses with a large number of tumor cells in some tissues, indicating that the lung tumor model was successfully constructed. Radon-exposed mice showed increased inflammatory cell infiltration, a higher count of tumor cells, and extensive congestion and necrosis in lung tissue compared to the control group. Radon exposure led to a marked increase in tumor burden in the lung tissues of KRAS^G12D^ mice due to a rise in tumor numbers and sizes (Figure 6E,F). In addition, Ki67 staining also showed that radon exposure promoted cell proliferation (Figure 6D,G). This suggests that radon exposure closely affects tumor progression in KRAS-driven lung cancer models. In many cases, functional changes in DNAm may regulate gene expression. To explore the link between radon exposure-induced DNAm and the expression of cancer-associated DMR-mapped genes, we examined their mRNA expression levels in the lung tissues of the radon-exposed and control groups (Figure 6H). We observed increased RNA expression levels of the hypomethylated gene PIK3R2, decreased RNA expression levels of the hypermethylated gene MAPK10, and increased RNA expression levels of the hypermethylated genes PLCG1 and PLCβ3 in radon-exposed KRAS^G12D^ mice. This difference may be due to the location where methylation occurs or the presence of other regulatory mechanisms interfering. Further, at the protein level, PIK3R2, PLCG1 and PLCβ3 were highly expressed and MAPK10 was poorly expressed in the lung tissue of radon-exposed KRAS^G12D^ mice (Figure 6I,J).

## 3. Discussion

Epigenetic regulation does not change gene sequences, but can affect physiological functions by regulating gene activity. Epigenetic changes may be reversible or transgenerational, which is crucial for the study of the association between environmental exposure (such as radiation exposure, including radon) and health [22,23]. In theory, epigenetic alterations induced by environmental exposure could be systemic (e.g., diffusion through the circulation or systemic inflammatory response), allowing epigenetic markers in accessible tissues (e.g., blood, saliva, etc.) to reflect changes in distant organs [24]. Recent studies have shown that radon exposure is associated with differential DNAm in human peripheral blood in terms of epigenetic mechanisms regulating gene expression [4,5], but it is not clear whether the changes induced by radon exposure in blood can be reflected in lung tissue changes. To explore whether there is consistency or correlation in DNAm episignatures in different tissues induced by radon exposure, we examined the differentially methylated DNA regions in lung tissue and blood of radon-exposed mice to assess whether this epigenetic marker can be used as an accessible biomarker for radon-induced lung tissue changes.

Overall, there were significant differences in DNAm patterns between the radon-exposed and control groups for both lung tissue samples and blood samples. To further identify DMRs related to radon exposure, lung tissue-derived DMRs and blood-derived DMRs had similar distribution proportions in chromosomes and gene functional regions. According to the GO and KEGG database analysis of the mapping genes of lung tissue-derived DMRs, radon exposure can affect cell proliferation, inflammatory response and cytoskeleton regulation by regulating PI3K-Akt, Hippo and other signaling pathways, and then induce lung inflammation, fibrosis or cancer. Previous studies have demonstrated that radiation-induced tissue specific damage can be reflected by blood detection by analyzing the methylation of circulating cell-free DNA (cfDNA). Similarly, in our study, alterations in DNAm in blood may also reflect the status of lung disease induced by radon exposure. Changes in the expression of Wnt, PI3K-Akt and other related genes in blood can reflect the abnormal proliferation and survival imbalance of lung cells induced by radon exposure [25,26]. Sphingolipid signaling and inflammatory mediators regulate changes in the TRP channel and other pathway genes, which reveal the inflammatory cascade and immune disorders induced by radon exposure [27,28,29,30,31]. Abnormalities in adrenergic, cholinergic synapses and proteoglycan related genes reflect the disease process of lung neural regulation disorder and extracellular matrix remodeling caused by radon exposure [32,33,34].

Radon exposure is significantly related to increased lung cancer risk [35,36]. Epidemiological data show that individuals residing in areas with concentrations exceeding 300 Bq/m^3^ face a notably higher risk of lung cancer as the exposure duration extends even beyond 40 years [37]. In this study, noteworthy alterations in DNAm levels in genes associated with the pathway in cancer were observed in lung tissue and blood samples after exposure to 2000 Bq/m^3^ of radon for 4 weeks. Considering the stability across microenvironment, clinical practicability and non-invasive advantages, and reflection of systemic characteristics of tumors, we focus on blood–lung overlap rather than lung-specific differentially methylated regions. Comparison of the differential DNAm positions between the exposed and control groups identified four DMRs showing consistent direction changes in lung tissue and blood samples: three hypermethylated DMRs located in the MAPK10, PLCG1 and PLCβ3 genes and one hypomethylated DMR located in the PIK3R2 gene. These findings suggest that the DNAm episignatures of the MAPK10, PLCG1, PLCβ3 and PIK3R2 genes may serve as potential blood biomarkers for radon-induced lung cancer.

The MAPK10 gene, also known as JNK3, is a serine/threonine protein kinase responsible for the regulation of cellular responses to various internal and external stimuli, such as stress, inflammation or cell injury [38,39]. Several studies have shown that abnormal activation or inhibition of MAPK10 signaling is associated with tumor cell proliferation, invasion and metastasis [40,41,42,43,44]. Notably, the expression of MAPK10 is associated with the incidence and progression of lung cancer [45,46,47], and heightened MAPK10 expression can enhance the effectiveness of chemotherapy on lung cancer [48]. In non-small cell lung cancer (NSCLC) tissues, MAPK10 expression is markedly reduced compared to healthy tissues, and it shows an inverse relationship with miR-21-5p. miR-21-5p targets the 3′-UTR region of MAPK10, thereby suppressing its expression, implying that MAPK10 may play a tumor suppressor role in lung cancer [49]. Ying et al. (2010) verified that methylation and silencing of MAPK10 gene are implicated in the advancement of B-cell lymphoma. Its tumor-specific methylation provides a promising molecular marker for diagnosis [50]. In this study, radon exposure induced MAPK10 5′-UTR hypermethylation, which was significantly correlated in lung tissues and blood, and its RNA and protein were significantly reduced in lung tissues of KRASG12D lung cancer mice. It has been found that MAPK10 is often downregulated by hypermethylation in its promoter region (including its 5′-UTR) region in HCC, and its methylation status is associated with a poor prognosis of patients. Demethylation treatment restored its expression, confirming that methylation affects gene expression by inhibiting transcription initiation [44]. Thus, MAPK10 gene hypermethylation within the 5′-UTR might silence gene expression, and disrupt the equilibrium between cell proliferation and apoptosis, resulting in aberrant signaling pathways, thereby representing a prospective biomarker and prime therapeutic target for radon-induced lung cancer.

PLCG1 encodes phospholipase Cγ1 (PLCγ1), and is pivotal in cellular signaling as it directly impacts receptor tyrosine kinases like VEGFR2. Elevated PLCG1 expression levels has been linked to tumor advancement and unfavorable prognosis [51,52,53]. According to Song et al. (2020), EphA2 receptor tyrosine kinase activates downstream signaling pathways by phosphorylating PLCγ1, which significantly promotes lung cancer cell proliferation and tumor growth [54]. Lu et al. (2020), by inhibiting the activity of PLCγ1 in lung adenocarcinoma cells, observed that the level of autophagy was significantly increased and led to cell death, further verifying the key role of PLCγ1 in maintaining tumor cell survival. Abnormal PLCγ1 expression has shown a positive correlation with the tumor stage and metastatic ability, supporting its potential as a target for lung cancer treatment [55]. Saliakoura et al. (2020) reported a significant connection between PLCγ1 expression levels and tumor cell metabolic adaptability, along with patient survival rates in a KRAS mutant lung adenocarcinoma model. Tumor cells with low expression of PLCγ1 show stronger hypoxia adaptation and higher proliferation activity, suggesting that elevated PLCγ1 expression may serve as a biomarker for poor NSCLC prognosis [56]. PLCG1 gene DNAm regulation within lung cancer remains unexplored. This study revealed radon exposure-induced PLCG1 hypermethylation within the intron region, correlating strongly across lung tissue and blood, and resulting in increased RNA and protein levels in the lung tissue of KRASG12D lung cancer mice. Introns, non-coding sequences within genes, through methylation (notably DNAm), play a crucial role in gene expression regulation [57]. For example, heightened intronic hypermethylation in the KRAS gene may continuously activate the MAPK signaling pathway, facilitating lung cancer cell proliferation via enhancer hijacking [58]. CDH1 (E-cadherin) intron methylation level increased in lung cancer tissues, links to greater invasiveness [59]. EGFR intron methylation level correlates positively with gefitinib resistance, possibly serving as a biomarker for targeted therapy effectiveness prediction [60]. It is speculated that PLCG1 intron methylation levels may assist in the early diagnosis or prognosis evaluation of lung cancer caused by radon exposure.

PLCβ3 belongs to the phospholipase C family. Prior research indicates that PLCβ3 transcripts are lost in certain tumor tissues and neuroendocrine tumor cell lines. When PLCβ3 is introduced into neuroendocrine cell lines that lack its expression, it can reduce tumor phenotypes and influence the gene regulation of human mismatch repair proteins, suggesting its potential role as a tumor suppressor in neuroendocrine tumorigenesis [61]. However, the function of PLCβ3 in tumors seems to be controversial in lung cancer. According to Zhang et al. (2019), elevated PLCβ3 mRNA levels correlate with poor overall survival rates in NSCLC patients, and correlates with poor prognosis in adenocarcinoma cases [62]. Limited research exists regarding PLCβ3 methylation. Fu et al. (2007) examined PLCD1, another enzyme from the PLC family, noting increased methylation in its promoter region’s CpG islands, causing reduced gene expression in various malignant tumors, such as esophageal cancer [63]. An extensive evaluation of DNAm status and gene expression in 12 tumors form the TCGA database revealed pronounced methylation across calcium signaling pathways, and genes in the key calcium signaling nodes showed notable hypermethylation [64]. As PLCβ3 interacts significantly with calcium signaling, it suggests that methylation may regulate PLCβ3 pathway in tumor [61]. In the present study, radon exposure induced PLCβ3 promoter hypermethylation, which was significantly correlated in lung tissue and blood, and its RNA and protein levels were significantly increased in lung tissue of KRASG12D lung cancer mice. This is inconsistent with the mechanism of gene silencing caused by promoter hypermethylation, implying alternate mechanisms besides DNAm could regulate PLCβ3 expression in lung cancer.

PIK3R2 serves as a regulatory component within the phosphatidylinositol-3-kinase (PI3K) complex. In lung cancer, PIK3R2 mutation may lead to the anomalous activation of the PI3K signaling pathway and fostered tumor progression and development [65,66]. Point mutations may alter protein functionality, like altering SH2 domain binding or lead to incessant PI3K signaling pathway activation, promoting tumor expansion and metastasis [67]. Analysis of data derived from the Cancer Genome Atlas (TCGA) public database revealed PIK3R2 expression in most tumors to be notably higher than in corresponding normal tissues, and this anomalous expression may be influenced by promoter methylation [68]. In the present study, radon exposure induced the hypomethylation of PIK3R2 3′-UTR, and its RNA and protein levels were significantly increased in lung tissues of KRASG12D lung cancer mice, though no significant correlation appeared between the methylation levels in lung tissue versus blood samples. Hypomethylation of the 3′-UTR region may alter the secondary structure of the mRNA, making it less amenable to nuclease recognition and degradation, thereby increasing the intracellular stability and half-life of PIK3R2 mRNA [69,70]. The 3′-UTR region is an important binding site of miRNA, which may be exposed or altered by hypomethylation. On the one hand, the miRNA that could bind to 3′-UTR and inhibit PIK3R2 expression could not bind properly, and the inhibitory effect on PIK3R2 expression was relieved [71]. On the other hand, new miRNA binding sites may be generated, recruiting some tumor-promoting miRNAs to bind to them and indirectly enhancing PIK3R2 expression. In addition, hypomethylation status may affect the binding of some proteins related to mRNA post-transcriptional processing and transport to 3′-UTR [72]. Accordingly, it is hypothesized that PIK3R2 3′-UTR hypomethylation may promote the development of lung cancer by enhancing mRNA stability and translation efficiency.

The current investigation is the first to analyze DNAm profiles in lung tissue under radon exposure, discovering DMRs in blood samples that correspond and correlate significantly with alterations observed in lung tissue samples. This study not only technically confirmed the accuracy of these DNAm episignatures, but also further verified the expression of DMRs-mapped genes concerning radon exposure using the GEMM. This examination provides new insights into mechanism driving radon-induced lung cancer and suggests potential targets for blood-based biomarkers and epigenetic treatment strategies. However, this study has limitations. We report hypermethylation and increased expression of certain genes, but it seems to contradict existing patterns of epigenetic regulation. Epigenetic regulation involves complex processes. Methylation ‘s impact on gene expression is not through absolute repression, but rather depends largely on sequence specificity, chromatin state, and interacting elements [73]. In future studies, the complex regulatory mechanisms between methylation and gene expression will be elucidated by further functional validation. While murine models provide mechanistic insights, human validation remains critical to establish the clinical utility of these episignatures. DNAm episignatures in radon-exposed murine models may translate to human populations through conserved epigenetic mechanisms and shared molecular pathways. Murine studies show radon-induced hypermethylation of tumor suppressor genes like RASSF1A [74], which is also aberrantly methylated in human lung cancer linked to radiation or smoking [75,76]. DNA repair and cell cycle regulatory pathways are conserved between mice and humans [77], suggesting that methylation changes in these pathways could serve as cross-species biomarkers. However, translation requires validation in human cohorts, particularly miners and environmentally exposed groups, to confirm dose-dependent effects and tissue-specificity. Epidemiological studies must also account for confounding factors like smoking and genetic background.

## 4. Materials and Methods

### 4.1. Mouse Models

C57BL/6N male mice, aged 6 weeks, were acquired from the SPF Biotechnology Co., Ltd. (Beijing, China). GEMMs, LSL-K-ras G12D mice, all in C57BL/6N background, were obtained from Cyagen Biotechnology Co., Ltd. (Suzhou, China). At 4 weeks old, mice underwent intratracheal infection with AVV-Cre (pAAV-CAG-EGFP-P2A-Cre-WPRE), supplied by the Obio Technology Co., Ltd. (Shanghai, China), at a concentration of 1 × 10^11^ plaque-forming units per mouse following a specified protocol [78]. Mouse were housed under controlled environmental conditions with a 12 h light/dark cycle, a room temperature of 22 ± 2 °C, and 50 ± 5% humidity. Details of the experimental modalities of this study are summarized in Table 3.

### 4.2. Radon Exposure

Mice were randomly assigned to either the radon exposure group (Rn group) or a control group (NC group). The experimental mice were housed in a radon chamber with a radon concentration of 2000 Bq/m^3^, along with decay products. This concentration was maintained consistently using an emanometer (NT8260, Jiangsu suhe instrument Co., Ltd., Suzhou, China). Radon exposure spanned 4 weeks, totaling up to 629 h, equivalent to an accumulated dose of 2 Working Level Months (WLM). Mice had unrestricted access to food and water during the exposure period. Mice of the control group were kept in a normal environment with a background radon level of 80 Bq/m^3^. For the GEMM studies, KRAS G12D mice were infected with AVV-Cre for 2 weeks before exposure to radon, alongside a control group.

### 4.3. Tissue Collection

Upon completion of the experiment, the mice were euthanized, and their lung tissues were excised, weighed, and either fixed in 4% paraformaldehyde for morphological analysis, or immediately frozen in liquid nitrogen for further analysis. Blood samples were stand at room temperature for half an hour, and blood cell samples were obtained by centrifugation (4°C, 10,000 rpm, 10 min), which were immediately flash frozen in liquid nitrogen and stored at −80 °C for DNA extraction and sequencing analysis.

### 4.4. Reduced Representation Bisulfite Sequencing (RRBS)

Genomic DNA was isolated from placental samples utilizing the Genomic DNA Kit (DP1101; BioTeKe Corpration, Beijing, China) following the supplier’s guidelines. To investigate genome-wide DNA methylation patterns in lung tissue and blood. RRBS was employed on samples from both radon-exposed and control groups. Each group included whole lung and blood samples from 6 mice, culminating in 24 samples overall. Initially, DNA samples were cleaved with methylation-insensitive restriction enzyme (MspI). Subsequently, DNA fragments underwent processes of 3′ end-repair, A′ tailing, and attachment of methylated adaptors. The Bisulfite treatment followed (EZ DNA Methylation Gold Kit, Zymo Research, Irvine, CA, USA), converting the unmethylated C to U (appearing as thymine post-PCR), while leaving methylated C intact. Finally, PCR was conducted, and the amplified porducts were pooled into a DNA library, which was then used for paired end sequencing to generate raw reads (HiSeq PE150; Illumina, San Diego, CA, USA).

### 4.5. Bioinformation Analysis

The analysis of differentially methylated regions (DMRs) was carried out using metilene software v0.2-7. The regions analyzed comprised at least five CpG sites with a minimum average methylation variance of 10% between groups, a maximum intersite distance of 200, and the number of NA in CPG sites per group was less than 20%. The software utilized a binary segmentation algorithm with combined statistical tests (MWU-test and 2D KS-test) to identify DMRs between the two groups. The mean methylation levels of each CpG site between radon exposed and control groups were compared and corrected for multiple testing, with differences reported as adj.*p* values. Sites with adj.*p* < 0.05 were considered DMRs between the groups. Average methylation levels (represented by adj.*p* value) of CpG sites from various genomic regions like upstream2k, exon, intron, downstream2k, CGI, CGI shore, and repeat regions were examined. Gene promoters were characterized as TSS 2000 (2000 bp upstream of the transcription start site). CpG contexts comprised island, shore regions, and others. A Manhattan plot illustrating probes locations on 19 chromosomes was generated using the CMplot package in R v4.4.2. The distributions of DMRs across chromosomes, gene structures, and CpG regions were documented. Gene Ontology (GO) and KEGG pathway analysis were conducted online (http://www.omicshare.com/, accessed on 6 December 2024) to determine potential functions/pathways involving differentially methylated genes. The top 15 enriched GO terms and pathways were ranked by adj.*p* value.

### 4.6. MassArray Methylation Analysis

To further validate RRBS findings, DMRs corresponding to genes associated with enriched pathways were selected for further validation in lung and blood samples. Target sequences were designed using the EpiDesigner 4.0 (Agena, San Diego, CA, USA) for primers specified in Table S5. The DNA isolation, quantification, identification, and bisulfite conversion processes mirrored previous descriptions. Assay DNA samples were prepared using the EpiTYPER™ Reagent Kit (Agena, San Diego, CA, USA) per the manufacturer’s protocol. Following bisulfite treatment, DNA samples underwent PCR amplification. PCR products were treated with shrimp alkaline phosphatase to eliminate free dNTP. Reverse transcription and enzymatic digestion were performed with T7 RNA and DNA polymerase and ribonuclease A. After resin purification, products were transferred to the SpectroCHIP matrix chip (Agena, San Diego, CA, USA). Post-sampling, the chip was analyzed by the MassARRAY Analyzer 4.0 (Agena, San Diego, CA, USA) and results were processed using EpiTYPER™ software v1.4.44 (Agena, San Diego, CA, USA).

### 4.7. Histology and Tumor Burden Analyses

Lungs were expanded via the tracheal injection of 4% paraformaldehyde, followed by a fixation process overnight. These specimens were then sent to Servicebio Technology Co., Ltd. (Wuhan, China) for paraffin embedding and slicing to a thickness of 5 μm. Slides were stained with hemotoxylin and eosin (H&E) and scanned using a slide scanner (Winmedic, Jinan, China, Win-20-S) for pathology assessment. Tumors were counted, and the tumor area percentage relative to the entire lung area was computed for each mouse to evaluate the tumor burden.

### 4.8. Immunohistochemistry (IHC)

Slides were deparaffinized and rehydrated. After cooling to room temperature, endogenous peroxidase activity was blocked, in accordance with the immunohistochemical staining kit (ZSGB-BIO, Shanghai, China, PV-6001). Sections underwent antigen retrieval by heating at 95 °C in citrate buffer with pH 6.0 for 10 min to reveal antigen surface sites. Blocking with 3% BSA for 1 h at room temperature, followed by an overnight incubation at 4 °C with Ki67 primary antibodies (1:200; Abcam, Cambridge, UK, ab15580). After application of secondary antibody for 1 h at room temperature, coloration was achieved through the DAB Peroxidase Substrate Kit (ZSGB-BIO, ZLI-9019). Hematoxylin was used for counterstaining, and slides were dehydrated and mounted for subsequent examination. Immunostained slides were digitized using a slide scanner (Winmedic, Belgrade, Serbia, Win-20-S), and Ki67-positive nuclei quantification within lung tumors was executed using Image J v2.9.0.

### 4.9. Real-Time Quantitative PCR (RT-qPCR)

Lung tissues RNA employing the TRIzol reagent (Invitrogen, Carlsbad, CA, USA) following standard protocols. Concentrations of the RNA sample were determined using the NanoDrop ND2000 instrument (Thermo, Waltham, MA, USA). cDNA synthesis was carried out with RT SuperMix for qPCR (Vazyme, Nanjing, China, R323) and RT-qPCR was conducted in triplicates using SYBR Mix (Vazyme, Nanjing, China, Q712). Detailed primer pair specifications are provided in Table S6.

### 4.10. Western Blots

Protein was extracted from lung tissues using protein extraction reagents (Thermo, Waltham, MA, USA, 78510), supplemented with inhibitors for protease and phosphatase activities. Following extraction, protein lysates were separated by SDS–polyacrylamide gel electrophoresis and transferred onto polyvinylidine difluoride membranes. Proteins were detected by standard Western blotting procedures, with antibody dilutions set at 1:1000 for PIK3R2 (Abcam, Cambridge, UK, ab180967), MAPK10 (Proteintech, Rosemont, IL, USA, 17572-1-AP), PLCG1 (Proteintech, Rosemont, IL, USA, 84941-4-RR), PLCβ3 (Proteintech, Rosemont, IL, USA, 66668-1-Ig) and β-actin (ZSGB-BIO, Beijing, China, TA-09). Imaging and quantification of bands were facilitated by Image J v2.9.0.

### 4.11. Statistical Analyses

Data are represented as mean ± SD. Student’s one-tailed *t*-test was applied for the comparison between two groups. Differences across groups were evaluated using one-way ANOVA. Spearman’s correlation examined the relationship between the DNAm levels of specific DMRs in lung versus blood samples. A significance threshold was set at *p* < 0.05. All computations were performed utilizing Prism version 8.0 (GraphPad Software Inc., San Diego, CA, USA).

## 5. Conclusions

The DNAm profiles in biological samples from the radon-exposed cohort diverged from those of controls. The lung tissue and blood samples exhibit similar methylation patterns. DNAm modifications of MAPK10, PLCG1, PLCβ3 and PIK3R2 genes were consistently linked between the lung and blood samples, suggesting their pivotal role in radon-induced lung cancer. Further extensive studies and population validation are required.

## Figures and Tables

**Table 1:** Lung-derived DNA methylation changes in cancer-related genes.

Gene	Chr	Start	End	Adj.*p*	Genome Region	CpG Context	Methylation
Tcf7	chr11	52,282,391	52,282,403	3.03 × 10^−4^	genebody; 5′-UTR	shore	Hyper
Smad3	chr9	63,667,363	63,667,524	2.19 × 10^−4^	genebody; exon; intron	other	Hyper
	chr9	63,737,702	63,737,925	5.83 × 10^−3^	genebody; intron	other	Hyper
Mapk10	chr5	103,144,437	103,144,464	1.60 × 10^−3^	genebody; 5′-UTR	other	Hyper
Plcg1	chr2	160,744,586	160,744,716	3.60 × 10^−3^	genebody; intron	other	Hyper
Gnas	chr2	174,300,082	174,300,258	5.31 × 10^−8^	genebody; 3′-UTR	Island; shore	Hyper
Plcb3	chr19	6,975,199	6,975,239	2.38 × 10^−11^	promoter; upstream 2k	shore	Hyper
Rassf5	chr1	131,199,252	131,199,429	8.22 × 10^−9^	genebody; intron	other	Hypo
Adcy7	chr8	88,288,484	88,288,659	3.60 × 10^−3^	genebody; 5′-UTR	other	Hypo
Pik3r2	chr8	70,768,477	70,768,628	2.57 × 10^−5^	genebody; 3′-UTR	Island; shore	Hypo

Abbreviations: Hyper, hypermethylation; Hypo, hypomethylation.

**Table 2:** Blood-derived DNA methylation changes in cancer-related genes.

Gene	Chr	Start	End	Adj.*p*	Genome Region	CpG Context	Methylation
Tcf7	chr11	52,283,626	52,283,697	2.90 × 10^−2^	promoter	other	Hyper
Mapk10	chr5	103,144,437	103,144,464	2.56 × 10^−4^	genebody; 5′-UTR	other	Hyper
Plcg1	chr2	160,744,586	160,744,716	1.85 × 10^−2^	genebody; intron	other	Hyper
Plcb3	chr19	6,975,199	6,975,239	7.49 × 10^−11^	Promoter	shore	Hyper
	chr19	6,955,776	6,955,844	4.03 × 10^−6^	genebody; exon	other	Hyper
Rassf5	chr1	131,187,911	131,188,029	1.95 × 10^−3^	genebody; intron	other	Hyper
	chr1	131,224,949	131,225,105	4.65 × 10^−4^	genebody; intron	other	Hypo
Adcy7	chr8	88,291,538	88,291,629	1.16 × 10^−2^	genebody; 5′-UTR	other	Hyper
Pik3r2	chr8	70,768,476	70,768,628	2.16 × 10^−2^	genebody; 3′-UTR	Island; shore	Hypo
Smad3	chr9	63,740,160	63,740,360	1.54 × 10^−2^	genebody; intron	other	Hypo

Abbreviations: Hyper, hypermethylation; Hypo, hypomethylation.

**Table 3:** Summary of the experimental patterns.

Experimental Group		Time Points		Sample Size	Purpose
		Exposure Period	Observation Period		
Wild type	Control	4 weeks	8 weeks	6	RRBS and MassArray
	Radon exposure	4 weeks	8 weeks	6	RRBS and MassArray
GEMM	Control	4 weeks	8 weeks	6	Expression validation
	Radon exposure	4 weeks	8 weeks	6	Expression validation

## Data Availability

The RRBS data have been deposited in the Sequence Read Archive (SRA) database under the accession number PRJNA1289886.

## Supplementary Materials

The following supporting information can be downloaded at: https://www.mdpi.com/article/10.3390/ijms26146873/s1.

## Abbreviations

The following abbreviations are used in this manuscript:

Rn	Radon
IARC	Agency for Research on Cancer
NSCLC	Non-small cell lung cancer
SCLC	Small cell lung cancer
DNAm	DNA methylation
TSGs	Tumor suppressor genes
ctDNA	Circulating tumor DNA
DMRs	Differentially methylated regions
PCA	principal component analysis
Hyper-DMR	Hypermethylated DMR
Hypo-DMR	Hypomethylated DMR
GO	Gene ontology
BP	Biological process
CC	Cellular component
MF	Molecular function
KEGG	Kyoto Encyclopedia of Genes and Genomes
cfDNA	Circulating cell-free DNA
PI3K	Phosphatidylinositol-3-kinase
TCGA	The Cancer Genome Atlas
WLM	Working Level Months
