# Supplementary material for: Characterization of DNA Methylation Episignatures for Radon-Induced Lung Cancer
Source: Int J Mol Sci. 2025 Jul 17;26(14):6873. doi: 10.3390/ijms26146873 (PMC12295219; doi:10.3390/ijms26146873)
Supplement: Supplementary file 1 [file ijms-26-06873-s001.zip › ijms-3741218-supplementary.pdf]

Supplementary Materials

Table S1. Most significant different DMR on chromosome 1 to 19 derived from mouse lung tissue.

ID	Chr	start	end	adj. *p*	Gene	Genome region	CpG context
dmr_10	chr1	159232632	159232678	6.99E-19	Rfwd2	genebody	Other
dmr_32	chr2	152707628	152707744	1.26E-24	H13	genebody; 3'-UTR	Island
dmr_103	chr3	58987290	58987326	1.38E-07	Rpl13-ps6	promoter; genebody; exon	Other
dmr_34	chr4	103325335	103325410	2.24E-10	Oma1	genebody; exon; intron	Other
dmr_36	chr5	107721058	107721220	1.79E-14	Gfi1	genebody; exon; intron	Island; shore
dmr_40	chr6	52203852	52204021	2.03E-22	Hoxa5; Hoxa3; Hoxaas3	genebody; exon; 5'-UTR	Island
dmr_43	chr7	82869786	82869891	2.97E-13	Mex3b	genebody; exon;	Island
dmr_78	chr8	105693815	105693880	1.86E-11	Rltpr	genebody; intron	Island
dmr_47	chr9	89066638	89067038	1.26E-24	Gm24463	promoter; genebody; Intron;	Island
dmr_2	chr10	127090960	127091145	9.24E-21	Agap2	genebody; exon; intron	Island
dmr_12	chr11	70520164	70520439	4.83E-29	Gltpd2	genebody; exon	Island
dmr_18	chr12	3235149	3235646	1.35E-17	Rab10os	promoter	Island
dmr_19	chr13	110399131	110399253	7.54E-12	Plk2	genebody; exon	Other
dmr_111	chr14	25304612	25304699	2.20E-09	Zmiz1os1	genebody; intron	Other
dmr_23	chr15	79895708	79895823	6.26E-18	Apobec3	genebody; intron	Other
dmr_26	chr16	92605946	92606118	1.97E-28	Runx1	genebody; exon	Island
dmr_52	chr17	13657302	13657363	2.98E-08	Gm41530	promoter	Other
dmr_28	chr18	74295000	74295230	2.72E-08	Cfap53	genebody; exon; intron	Other
dmr_31	chr19	7423402	7423836	3.60E-14	Mir6991; 2700081O15Rik	genebody; 3'-UTR; downstream 2k	Island

Table S2. Most significant different DMR on chromosome 1 to 19 derived from mouse peripheral blood.

ID	Chr	start	end	adj. *p*	Gene	Genome region	CpG context
dmr_6	chr1	159232402	159232459	1.49E-20	Rfwd2	genebody; 5'-UTR	Island
dmr_20	chr2	145860554	145860772	1.70E-08	Rin2	genebody; exon	Island
dmr_40	chr3	88615949	88616090	3.40E-08	Arhgef2; Gm20652	genebody;intron; downstream 2k	Other
dmr_21	chr4	139556032	139556254	4.35E-09	Iffo2	genebody; 5'-UTR	Other
dmr_23	chr5	136102299	136102461	9.85E-25	Rasa4	genebody; exon; intron	Island
dmr_26	chr6	10690652	10690898	5.64E-18	-	-	-
dmr_27	chr7	4478182	4478491	9.17E-12	Eps8l1	genebody; exon; intron	Other
dmr_33	chr8	88627384	88627507	1.28E-49	Snx20	genebody; exon; intron	Island
dmr_34	chr9	89066628	89066987	3.05E-30	Gm24463	promoter; genebody; Intron	Island
dmr_2	chr10	4712369	4712608	2.80E-15	Esr1	genebody; 5'-UTR	Island
dmr_5	chr11	12026388	12026469	7.16E-18	Grb10	genebody; 5'-UTR	Other
dmr_12	chr12	3235282	3235566	1.52E-15	Rab10os	promoter	Island
dmr_35	chr13	110564336	110564485	2.82E-07	-	-	-
dmr_13	chr14	33189867	33190085	8.24E-16	Wdfy4	genebody; 5'-UTR	Other
dmr_32	chr15	25364239	25364419	4.12E-28	Basp1	genebody; exon	Island
dmr_53	chr16	25220574	25220645	1.19E-09	-	-	-
dmr_16	chr17	87745913	87746208	3.61E-21	Kcnk12	genebody; 3'-UTR	Island; shore
dmr_18	chr18	30267932	30268177	8.57E-15	-	-	-
dmr_19	chr19	6975199	6975239	7.49E-11	Ppp1r14b; Plcb3	promoter; genebody; exon	Shore

Table S3. Top 15 significantly GO terms.

Term	Class	Name	Gene number	Foldchange	adj.*P*
Lung					
GO:0003677	molecular_function	DNA binding	96	2.899114	2.25E-15
GO:0046872	molecular_function	metal ion binding	167	2.957579	5.42E-27
GO:0005515	molecular_function	protein binding	292	3.378544	1.60E-55
GO:0030054	cellular_component	cell junction	60	4.408897	7.63E-17
GO:0005856	cellular_component	cytoskeleton	78	3.691695	1.11E-17
GO:0070062	cellular_component	extracellular exosome	132	2.647732	8.57E-18
GO:0005886	cellular_component	plasma membrane	223	3.179721	7.04E-40
GO:0005634	cellular_component	nucleus	259	2.326505	5.09E-26
GO:0005737	cellular_component	cytoplasm	333	2.671595	5.38E-43
GO:0016020	cellular_component	membrane	368	2.763395	9.36E-50
GO:0000122	biological_process	negative regulation of transcription by RNA polymerase II	70	5.093072	9.39E-23
GO:0007275	biological_process	multicellular organism development	74	3.760411	3.36E-17
GO:0006351	biological_process	transcription, DNA-templated	106	3.001364	8.57E-18
GO:0006355	biological_process	regulation of transcription, DNA-templated	109	2.77631	3.82E-16
GO:0006468	biological_process	protein phosphorylation	51	4.661279	3.14E-15
Blood					
GO:0016740	molecular_function	transferase activity	60	3.412839	3.90E-12
GO:0005524	molecular_function	ATP binding	60	3.595625	5.83E-13
GO:0000166	molecular_function	nucleotide binding	70	3.085614	3.83E-12
GO:0046872	molecular_function	metal ion binding	104	2.908177	2.62E-16
GO:0005515	molecular_function	protein binding	165	3.014382	9.15E-27
GO:0005856	cellular_component	cytoskeleton	47	3.512341	5.69E-10
GO:0016021	cellular_component	integral component of membrane	137	2.090072	1.06E-10
GO:0005886	cellular_component	plasma membrane	143	3.219495	4.93E-26
GO:0005634	cellular_component	nucleus	168	2.382765	1.41E-17
GO:0016020	cellular_component	membrane	251	2.976025	5.66E-38
GO:0005737	cellular_component	cytoplasm	219	2.774202	5.95E-30
GO:0035556	biological_process	intracellular signal transduction	27	5.83433	5.81E-10
GO:0006468	biological_process	protein phosphorylation	34	4.90661	1.31E-10
GO:0000122	biological_process	negative regulation of transcription by RNA polymerase II	40	4.595253	1.19E-11
GO:0007165	biological_process	signal transduction	51	3.42575	1.90E-10

Table S4. Top 15 significantly KEGG pathway.

Term	Name	Gene number	Foldchange	adj.*P*
Lung				
mmu05200	Pathways in cancer	37	4.870354	1.86E-11
mmu04530	Tight junction	21	6.505103	1.66E-08
mmu04015	Rap1 signaling pathway	22	5.343114	1.12E-07
mmu04071	Sphingolipid signaling pathway	17	7.208427	1.12E-07
mmu04611	Platelet activation	16	6.674974	7.11E-07
mmu04072	Phospholipase D signaling pathway	17	5.982503	9.15E-07
mmu04022	cGMP-PKG signaling pathway	18	5.542614	9.15E-07
mmu04810	Regulation of actin cytoskeleton	20	4.834678	9.43E-07
mmu04390	Hippo signaling pathway	17	5.710571	9.43E-07
mmu04916	Melanogenesis	14	7.242347	9.43E-07
mmu04933	AGE-RAGE signaling pathway in diabetic complications	14	7.242347	9.43E-07
mmu04360	Axon guidance	18	5.320908	9.43E-07
mmu04510	Focal adhesion	19	4.939147	1.11E-06
mmu04151	PI3K-Akt signaling pathway	25	3.737793	1.59E-06
mmu04310	Wnt signaling pathway	16	5.669157	1.87E-06
Blood				
mmu04261	Adrenergic signaling in cardiomyocytes	16	8.89041	6.12E-08
mmu04310	Wnt signaling pathway	14	7.832387	1.73E-06
mmu04728	Dopaminergic synapse	12	7.314683	2.39E-05
mmu04725	Cholinergic synapse	11	7.951211	2.58E-05
mmu04360	Axon guidance	13	6.067703	2.60E-05
mmu05205	Proteoglycans in cancer	14	5.55111	2.60E-05
mmu04915	Estrogen signaling pathway	10	8.508401	2.60E-05
mmu04071	Sphingolipid signaling pathway	11	7.364648	2.60E-05
mmu04750	Inflammatory mediator regulation of TRP channels	11	7.187893	2.73E-05
mmu04916	Melanogenesis	10	8.168059	2.73E-05
mmu04911	Insulin secretion	9	8.648532	5.49E-05
mmu04925	Aldosterone synthesis and secretion	9	8.547975	5.50E-05
mmu04151	PI3K-Akt signaling pathway	17	4.01321	6.24E-05
mmu04670	Leukocyte transendothelial migration	10	7.102661	6.24E-05
mmu04072	Phospholipase D signaling pathway	11	6.112157	7.11E-05

Table S5. The primer sequences for amplifying target genes in MassArray methylation.

Gene name	Forward primer (5'-3')	Reverse primer (5'-3')
PLCG1	aggaagagagTTTTTGGTATAGGTGT AGGTGGTTT	cagtaatacgactcactatagggagaaggctTCCAAAATACC CAAAAACCCTAATA
PIK3R2	aggaagagagTTTTTGAAGGAGTTG GTGTTGTATT	cagtaatacgactcactatagggagaaggctCCAAACCTAAA TTCCTAAAAACCA

Table S6. The primer sequences for qRT-PCR.

Gene name	Forward primer (5'-3')	Reverse primer (5'-3')
MAPK10	AGACATGCAGTGAAAGGCGA	TTTGCTGCCCAGAGAGAGTG
PLCG1	AGCTACAACGTGTACAGTCCC	TGTCCTTGACCCACACTCTG
PLCβ3	AGCTCTTTGCACCCGAGTTT	AATAGCCAGGACTGGGACGA
PIK3R2	CACTGACAGCCTTTCTTCGG	GGGTTCCCCAATAACCCCAA
β-actin	TGCTGTCCCTGTATGCCTCT	TTTGATGTCACGCACGATTT

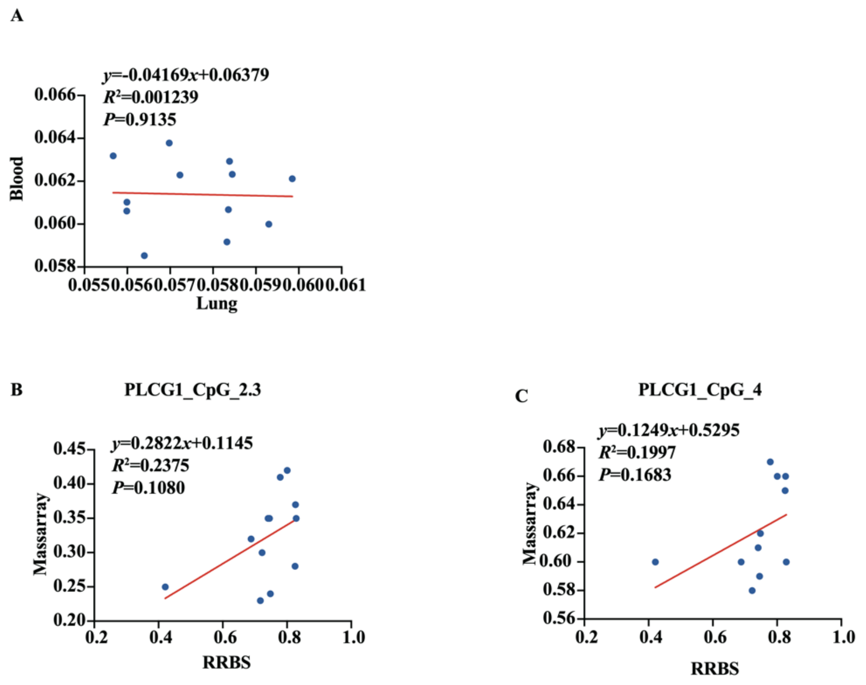

Figure S1. Consistency analysis of methylation in different tissues or detection techniques. (A) Scatter plots showing the correlation of methylation in lung and blood measured by RRBS. (B) Scatter plot shows the correlation of methylation level in PLCG1 target region in blood measured by RRBS (X axis, methylation level in PLCG1_Chr2_160744586_160744716) and MassArray (Y axis, methylation level in PLCG1 CpG 2.3 site). (C) Scatter plot shows the correlation of methylation level in PLCG1 target region in blood measured by RRBS (X axis, methylation level in PLCG1_Chr2_160744586_160744716) and MassArray (Y axis, methylation level in PLCG1 CpG 4 site).
